# Radiation Exposure from Diagnostic Imaging in a Cohort of Pediatric Transplant Recipients

**DOI:** 10.1371/journal.pone.0167922

**Published:** 2017-01-12

**Authors:** Alexandra Seal, Michael Hawkes, Ravi Bhargava, Michelle Noga, Jutta Preiksaitis, Curtis Mabilangan, Joan Robinson

**Affiliations:** University of Alberta and Stollery Children’s Hospital, Edmonton, Alberta, Canada; Northwestern University Feinberg School of Medicine, UNITED STATES

## Abstract

Recipients of solid organ transplants (SOT) have extensive diagnostic imaging (DI). The purpose of this study was to quantify this exposure. Children from northern Alberta with SOTs at Stollery Children’s Hospital, Edmonton, Alberta January 1, 2006, to July 31, 2012, were included. Effective doses of radiation were estimated using published norms for DI performed post-transplant up to October 16, 2014. The 54 eligible children had 6215 DI studies (5628 plain films, 293 computerized tomography (CT) scans, 149 positron emission topography (PET) -CT scans, 47 nuclear medicine scans and 98 cardiac catheterizations). Children less than 5 years of age underwent more DI studies than did older children (median (IQR) 140 (66–210) *vs* 49 (19–105), p = 0.010). Children with post-transplant lymphoproliferative disorder (N = 8) had more CT scans (median (IQR) 13 (5.5–36) *vs* 1 (0–5), p<0.001) and PET-CT scans (median (IQR) 3.5 (1.5–8) *vs* 0 (0–0), p<0.001) than did other children. The estimated cumulative effective dose attributed to DI studies post-transplant was median (range) 78 (4.1–400) millisievert (mSv), and 19 of 54 children (35%; 95% confidence interval 24–49%) had a dose >100 mSv. In conclusion, a significant proportion of pediatric transplant recipients have sufficient radiation exposure post-transplant for DI to be at potential risk for radiation-induced malignancies.

## Introduction

The risk of malignancy may be increased by exposure to radiation for diagnostic imaging (DI) [1]. One study estimated that 1% of malignancies in Canada and the United States (US) are attributable to radiation from DI [2]. The maximum permissible occupational radiation exposure in the US is 50 millisievert (mSv) per year or 100 mSv over 5 years [3] while background radiation is about 3 mSv annually [4]. The lifetime excess risk of death from cancer has been estimated to increase by approximately 0.4% with 100 mSv of cumulative radiation exposure [5] and to be 5% per sievert [6]. It is controversial whether cumulative doses below 100 mSv increase the risk of malignancy [7]. A further theoretical concern is that detrimental genetic effects may result from high cumulative doses of radiation from DI to the gonads.

Pediatric solid organ transplant (SOT) recipients typically have multiple DI studies for diagnosis and management of their underlying disorder, for peri- and post-operative care, and for diagnosis and management of complications. This DI often includes computerized tomography (CT) and other modalities that typically involve much higher doses of ionizing radiation than do plain radiographs. Children who develop Epstein Barr virus (EBV) DNAemia post-transplant are at increased risk of post-transplant lymphoproliferative disorder (PTLD) and often have extensive DI to diagnose and manage PTLD.

A previous study reported the short-term radiation exposure from DI of children in the intensive care unit measured using dosimeters [8] but there appear to be no published studies of long-term exposure for children with chronic diseases. The primary purpose of this study was to describe the exposure from DI for pediatric SOT recipients post-transplant. Secondary outcomes were the correlation between the amount of radiation and a) transplant organ and b) the presence or absence of EBV DNAemia and PTLD.

## Patients and Methods

Ethics approval was obtained for this cohort study from the Health Research Ethics Board of the University of Alberta. Parental consent was waived and data were anonymized prior to analysis. A list of all solid organ transplants (SOTs) in children up to 18 years of age performed at the Stollery Children’s Hospital from January 1, 2006 to July 31, 2012 was obtained from Transplant Services. Data on EBV DNAemia were obtained from the Alberta Provincial Laboratory for Public Health laboratory information system and presence or absence of PTLD from a prospective PTLD database. The frequency of testing for EBV is determined by a protocol based on donor and recipient EBV status.

### Inclusion criteria

All SOT recipients living in northern Alberta (from Red Deer north) at the time of transplant were enrolled in the study with the only exclusion criteria being death within the first year post-transplant. The Stollery Children’s Hospital is the only tertiary care pediatric hospital in northern Alberta, the only hospital in the province that performs SOTs and the primary referral center for pediatric thoracic and liver transplant in Western Canada. Therefore, one would anticipate that almost all DI for SOT recipients living in northern Alberta would be performed at the Stollery Children’s Hospital.

### Data collection

The Picture Archive and Communication System (PACS) at the Stollery Children’s Hospital has records of all plain films and CT scans performed during the study period. A small number of studies could not be included in the current study as nuclear medicine results were first added to PACS March, 2007 and cardiac catheterization data April, 2008. PACS was searched for all included children and the number of studies of each type from the date of transplant up to October 16, 2014 was recorded. Imaging studies were classified based on technique and location into the following categories: abdominal radiographs, chest radiographs, head and neck radiographs, extremity radiographs, CT chest, CT abdomen, CT pelvis, CT head, PET (positron emission topography) combined with CT and nuclear imaging. For plain films, each view was counted as an examination. For patients who had more than one transplant during the study, data were collected from the date of the first transplant.

The estimated effective dose of radiation for CT scans of the head (2.68 mSv) and abdomen (5.06 mSv) [9] and PET-CT scans (10 mSv) [10] were based on recently published estimated pediatric doses. A dose of 5 mSV was estimated for each cardiac catheterization, also based on a recent pediatric publication [11] Effective doses of radiation for all other studies including CT scans of the CT chest (7 mSv), neck (3mSv) and pelvis (6 mSV) were estimated using a standard adult catalog [12] as there are no well-established pediatric estimates. Doses were not corrected for the year of the study or the age or gender of the child as there is no accepted published methodology for doing so.

### Statistical analysis

The median number of each imaging type and the estimated effective radiation dose were calculated for the first year post-transplant and then for all subsequent years since one would anticipate far less DI after the first year. We calculated annual rates in the first and subsequent years after transplantation to account for possible effects of differential follow-up time. Data were calculated for the entire study population and then according to transplant organ, age (arbitrarily divided into < 5 years versus ≥ 5 years), and presence or absence of EBV DNAemia and of PTLD. Non-Gaussian distribution of count data and radiation doses were observed; therefore summary statistics were expressed as median with range or inter-quartile range. Comparative statistics employed non-parametric methods (Mann-Whitney U-test, Kruskall-Wallis test, Spearman’s rank correlation coefficient (ρ) and test). Analysis was performed with GraphPad Prism6 (GraphPad Software Inc., La Jolla, CA) and IBM SPSS Statistics 19 (SPSS Inc., Chicago, IL).

Our primary question was the proportion of children who received a radiation dose >100mSv. Using standard sample size calculations, and assuming that a proportion of 25% of children would receive a high radiation dose (>100 mSv), we estimated that we would need at least 41 patients to estimate this proportion to within +/- 15% (95% confidence interval).

## Results

### Study subjects

There were 292 pediatric SOTs performed during the study period of which 55 lived in northern Alberta at the time of the transplant. One liver transplant recipient died 3 days post-transplant so was excluded from the analysis. The final cohort consisted of 54 children (30 females and 24 males) who were followed for a median of 6.0 years (interquartile range (IQR) 4.0–7.5 years), for a total of 312 patient-years of follow-up. Forty-seven of these 54 children had one transplant and 7 had two transplants during the study period. The median age at the time of transplant was 7.7 years (range 2 months to 16 years) for single transplant recipients. Children who received more than one transplant during the study received their first transplant at a median age of 1 year (range 6 months to 15 years) and received their second transplant after a median interval of 9 days (range 1 day to 3 years). Three children included in the study died during the study period at 2.4, 2.8 and 4.0 years post-transplant.

### DI studies performed

**Fig 1:**
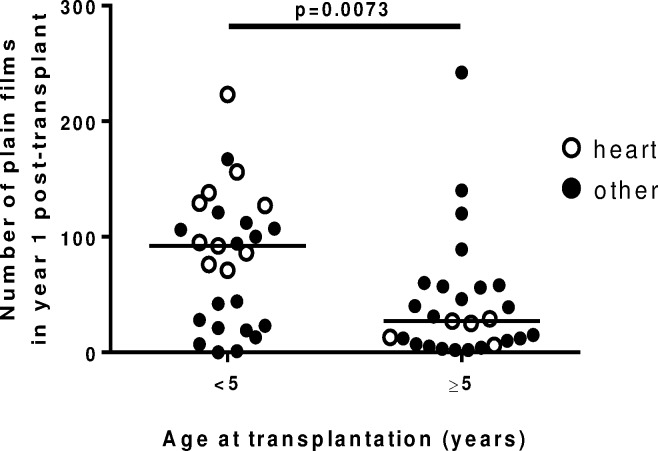
Number of plain films in first year post-transplant by age in 54 SOT recipients. White circles represent heart transplant recipients, who underwent the highest number of plain film studies. Horizontal lines represent the median number of imaging studies in each subgroup.

A total of 6215 DI studies (5628 plain films, 293 CT scans, 149 PET-CT scans, 47 nuclear medicine scans and 98 cardiac catheterizations were performed on this cohort of 54 patients. This represents a median of 82 studies per patient (range 5 to 527), with 10 of 54 patients (19%) undergoing more than 200 DI studies. The annual number of studies was median (IQR) 51 (14–110) in the first year post-transplant and 4 (1–15) per year in subsequent years. Children less than 5 years of age underwent significantly more DI studies than did older children (median (IQR) 140 (66–210) *vs* 49 (19–105) (p = 0.010). This difference was driven mainly by a higher number of plain films in the first year post-transplant among young heart transplant recipients (Fig 1).

**Fig 2:**
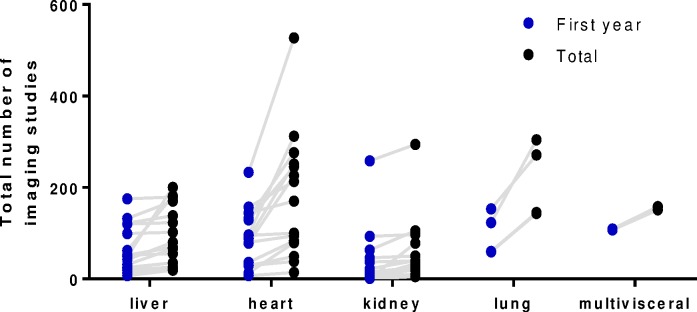
Total number of diagnostic imaging studies in 54 SOT recipients by organ type over a median of 6 years post-transplant. Horizontal lines represent the median number of imaging studies in each subgroup.

Table 1 shows the number of imaging studies disaggregated by transplant organ type and Fig 2 shows the distribution. Significant differences in the numbers of plain films (highest among heart and lung transplant recipients) (p<0.001) as well as nuclear medicine scans (highest among kidney transplant recipients) (p<0.001) were observed.

**Table 1:** Number of imaging studies per child, disaggregated by transplant organ type reported as median (IQR) unless otherwise specified.

	Liver (n = 18)	Heart (n = 15)	Kidney (n = 15)	Lung (n = 4)	Multivisceral (n = 2)*	p-value
***Year 1***						
** Plain film**	41 (13–96)	86 (28–128)	10 (2–40)	89 (56–135)	106, 107	0.006
** Cardiac cath**	0 (0–0)	2 (1–3)	0 (0–0)	0 (0–0)	0, 0	<0.001
** CT**	1 (0–6)	0 (0–2)	0 (0–1)	2 (1–6)	2, 0	0.18
** PET**	0 (0–0)	0 (0–0)	0 (0–0)	0 (0–2)	0, 0	0.37
** NM**	1 (0–1)	0 (0–0)	3 (2–5)	2 (1–3)	1, 0	<0.001
***Subsequent years***						
** Plain film**	7 (3–18)	44 (11–133)	3 (2–27)	97 (80–160)	48, 28	0.003
** Cardiac cath**	0 (0–0)	4 (4–6)	0 (0–0)	0 (0–0)	0, 0	<0.001
** CT**	1 (0–2)	0 (0–5)	0 (0–1)	4 (4–6)	0, 13	0.084
** PET**	0 (0–0)	0 (0–0)	0 (0–0)	0 (0–0)	0, 3	0.63
** NM**	1 (0–2)	1 (0–2)	2 (1–2)	1 (0–3)	1, 0	0.43
***Total***						
** Plain film**	56 (18–120)	158 (69–225)	21 (11–75)	195 (140–280)	154, 135	0.002
** Cardiac cath**	0 (0–0)	7 (5–9)	0 (0–0)	0 (0–0)	0, 0	<0.001
** CT**	3 (1–7)	2 (0–5)	1 (0–2)	7 (5–10)	2, 13	0.087
** PET**	0 (0–1)	0 (0–1)	0 (0–0)	0 (0–2)	0, 3	0.76
** NM**	2 (1–4)	1 (0–2)	4 (2–7)	3 (2–5)	2, 0	<0.001
**TOTAL studies**	68 (24–150)	170 (79–250)	33 (19–78)	208 (140–300)	158, 151	0.002
**TOTAL effective radiation dose [mSv]**	54 (26–130)	81 (55–140)	48 (14–100)	130 (53–270)	78, 118	0.16

* With 2 patients in this category, the values for both patients are provided, instead of median (IQR).

Legend: cath–catheterization; CT—computerized tomography studies; mSv—millisievert; NM–nuclear medicine studies; PET—positron emission topography studies

### Relationship between EBV DNAemia, PTLD status and number of DI studies

**Fig 3:**
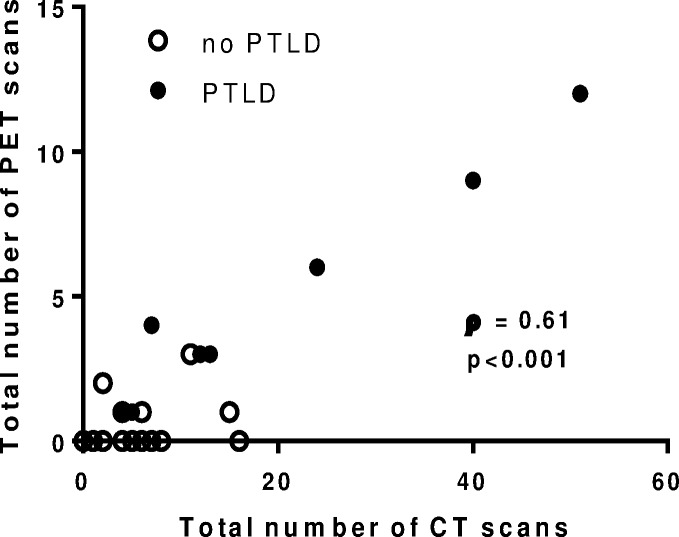
Number of CT and PET-CT scans in 54 SOT recipients over a median of 6 years post-transplant according to PTLD status. Solid circles represent patients with PTLD, who underwent the most CT and PET-CT scans.

EBV DNAemia was detected during the study period in 32 of the 54 children (59%) and was detected in the first year post-transplant in 18 of these 32 children. PTLD was diagnosed in 8 of the 54 patients (15%) with 2 of the 8 cases being diagnosed in the first year post-transplant. All patients with PTLD also had EBV DNAemia. Table 2 shows the number of imaging studies disaggregated by EBV DNAemia and PTLD diagnosis. The number of CT scans and PET-CT scans performed beyond the first year post transplant and the total number of CT scans and PET-CT scans were statistically significantly higher among patients with PTLD than among those without PTLD (p<0.001 for all comparisons). These differences are unlikely to be due to different duration of follow-up, since median (IQR) follow-up period was similar between groups [6.4 (3.9–7.8), 5.9 (3.7–7.7), 5.4 (3.2–6.7) for no EBV DNAemia, EBV DNAemia, and PTLD, respectively, p = 0.50]. Nonetheless, to account for possible inequalities in follow-up time between groups, the annual rate of CT scans and PET-CT scans beyond year 1 post-transplant was compared and was significantly different between groups (p<0.001 for both comparisons). There were no significant differences in the number of CT scans and PET-CT scans between patients with EBV DNAemia but no PTLD, and patients without EBV DNAemia (p>0.05 for all comparisons). There was a positive correlation between the number of CT scans and the number of PET-CT scans, with PTLD patients undergoing the highest number of both modalities (Spearman’s ρ = 0.61, p<0.001, Fig 3).

**Table 2:** Number of imaging studies, disaggregated by EBV DNAemia and PTLD, median (IQR) unless otherwise specified.

	No EBV (n = 22)	EBV DNAemia (n = 24)^1^	PTLD^1^^,^^2^ (n = 8)	p-value
***Year 1***				
** Plain film**	30 (6–96)	40 (13–120)	73 (43–99)	0.34
** Cardiac cath**	0 (0–1)	0 (0–0)	0 (0–1)	0.91
** CT**	1 (0–2)	0 (0–4)	0 (0–4)	0.95
** PET**	0 (0–0)	0 (0–0)	0 (0–1)	0.20
** NM**	1 (0–2)	1 (0–3)	0 (0–3)	0.71
***Subsequent years***				
** Plain film**	9 (2–80)	12 (4–33)	30 (2–110)	0.81
** Cardiac cath**	0 (0–4)	0 (0–0)	0 (0–3)	0.71
** CT**	0 (0–1)	0 (0–2)	13 (5–27)	<0.001
** PET**	0 (0–0)	0 (0–0)	4 (2–7)	<0.001
** NM**	1 (0–2)	2 (0–3)	1 (0–1)	0.26
***Total***				
** Plain film**	73 (15–140)	56 (20–170)	120 (54–200)	0.40
** Cardiac cath**	0 (0–6)	0 (0–0)	0 (0–4)	0.57
** CT**	1 (0–4)	2 (0–7)	13 (6–36)	0.001
** PET**	0 (0–0)	0 (0–0)	4 (2–8)	<0.001
** NM**	2 (1–3)	3 (1–6)	2 (0–4)	0.32
**TOTAL studies**	81 (19–150)	68 (25–180)	160 (61–230)	0.21
**TOTAL effective radiation dose [mSv]**	78 (38–120)	60 (25–120)	120 (38–200)	0.43

^1^EBV DNAemia was detected in the first year post-transplant in 14/24 (58%) of cases. Two of the 8 PTLD cases were diagnosed in the first year of life

^2^All patients with PTLD also had EBV DNAemia

### Cumulative radiation exposure

**Fig 4:**
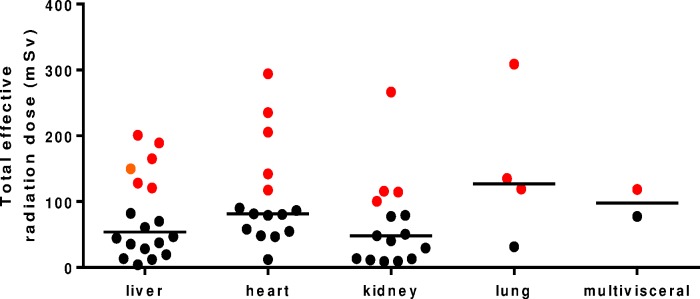
Cumulative effective radiation dose in 54 SOT recipients over a median of 6 years post-transplant. Solid red circles represent patients who received an effective radiation dose > 100 mSV, a threshold associated with increased cancer risk. Horizontal lines represent the median effective dose in each subgroup.

Total estimated cumulative effective dose of radiation attributed to DI studies post-transplant was median (range) 78 (4.1–400) mSV and 19 of 54 children (35%; 95% confidence interval 24–49%) had a dose >100 mSv with at least one child of every transplant organ type meeting this criteria (Fig 4). Tables 1 and 2 and Fig 3 show the total effective radiation dose disaggregated by transplant organ type and EBV/PTLD status.

## Discussion

This study showed that pediatric SOT recipients had a large number of DI studies performed post-transplant with those who were less than 5 years of age at the time of SOT having more studies than older recipients. About one in three children had a cumulative effective dose of radiation above 100 mSv, a level that is considered to increase the risk of radiation-induced malignancy [7]. Children with PTLD were at particularly high risk with a median of 12 CT scans and 4 PET-CT scans per child over the study period.

In a similar study from The Hospital for Sick Children in Toronto (described in only an abstract to date), 524 SOT recipients had a lower number of total DI studies (a median of 38 per child over a median of 3 years versus 51 per child in just the first year in the current study) and a lower median cumulative radiation exposure (6 mSv over median 3 years versus 43 mSv over median 6 years in the current study) with only 3% receiving > 100 mSv (versus 22% in the current study) [13]. The differences in the estimates between the two studies may be partially accounted for by the fact that there are no reference standards for pediatric radiation doses and they presumably used lower estimated doses. In the current study, had we used the estimated doses for our current CT scanner (1.5mSv for head and 2 mSv for abdomen) rather than the estimates from the literature (2.68 mSv for head and 5.06 mSv for abdomen) [9], the total estimated cumulative effective dose of radiation attributed to DI studies post-transplant fell from median (range) 78 (4.1–400) mSV to median (range) 43 (4.1–380) mSv and the number of children with a dose > 100 mSv fell from 19 of 54 children (35%; 95% confidence interval 24–49%) to 12 of 54 children (22%; 95% confidence interval 13–35%).

There are three studies in adults with similar methodology. A mean of 161 DI studies and 138 mSv effective radiation exposure were documented during a mean of 6.5 years follow-up for 107 lung transplant recipients [3]. A study of 202 heart transplant recipients followed for up to 10 years reported a mean of 66 DI studies and 84 mSv effective radiation exposure post-transplant [14]. The first year accounted for about 40% of the total radiation dose (versus 56% in the current study). Although the adults in both studies received a higher mean dose of radiation than in the current pediatric study, the authors of these studies concluded that this mean dose increased the risk of cancer by only 0.55% [3] and 0.34% [14] respectively so was of minimal concern. The third study included kidney transplant patients and showed that their mean annual dose of radiation from DI (16.3 mSv) was in the same range as that for patients on hemodialysis not being considered for transplant (18 mSv) [15], demonstrating that patients with chronic illness often have extensive DI with or without a transplant.

Nonetheless, there are reasons to be particularly concerned about young children and extensive radiation exposure from DI. They have many more years of both exposure and risk of malignancy than do adults post-transplant. The cancer risk in male infants is postulated to potentially be three to four fold that of men aged 20 to 50 years exposed to the same radiation dose with female infants having double the risk of males [16]. It seems plausible that immunosuppression and radiation exposure could be synergistic in increasing the risk of malignancy over a span of decades. In particular, clinicians should be cautious about repeated use of PET-CT in children with possible PTLD. European guidelines recommend routine use in asymptomatic patients with a ten-fold rise in EBV DNAemia or a persistent high level with mononucleosis-like disease [17]. There is no evidence that this improves outcomes. There is a need to compare the sensitivity of careful clinical palpation of the neck combined with a chest x-ray and ultrasound or magnetic resonance imaging of the abdomen and pelvis versus PET-CT for diagnosis of PTLD in children with EBV DNAemia and non-specific clinical signs. Diffusion weighted imaging with body suppression involves no radiation and may prove to be useful for diagnosis and follow-up of PTLD.

Ideally, one would perform a prospective study that includes DI prior to SOT. Previous studies have documented high doses of radiation in adults awaiting kidney transplant with 30% having >50mSv radiation for investigations done solely for the transplant work-up [3]; however, the bulk of the radiation in this study was for nuclear stress tests which would rarely be performed in children awaiting transplant.

The primary limitation of the current study is that as in previous studies [18], doses of radiation were derived from the literature rather than being subject-specific. There are no published pediatric norms for many types of imaging so adult data had to be used. Use of contrast and fluoroscopy was not factored in as it is not clear how to do so accurately. It would be optimal to include pre-transplant DI but this was not practical as children may not have lived in northern Alberta right from birth. Determining the actual dose of radiation by having children with chronic diseases where a dosimeter during all DI studies would be ideal. Data on the radiosensitivity of different tissues and of males versus females is an evolving field but ideally would be factored in when estimating the risk of harm from DI [16].

Another limitation is that records of DI performed outside of the Stollery Children’s Hospital were not available. It seems likely that almost all studies involving significant radiation would have been performed at the Stollery Children’s Hospital with only occasional chest radiographs or plain films following trauma performed in smaller hospitals. Study subjects could have lived outside of northern Alberta for some of the time post-transplant. As mentioned in the methods, nuclear medicine scans performed between January 2006 and March 2007 and cardiac catheterizations performed between Jan 2006 and April 2008 could not be included in the study. A further limitation was that because of the small sample size, EBV DNAemia was only examined qualitatively rather than analyzing the peak viral load, the rate of rise in the viral load or the time of onset of EBV DNAemia or PTLD.

Efforts must be made to ensure that those ordering DI for SOT recipients appreciate the large number of studies ordered on these children. All should follow the ALARA (As Low As Reasonably Achievable) radiation safety principle by maximizing use of ultrasound and magnetic resonance imaging and limiting studies with ionizing radiation to those that are likely to alter patient management.
